# The Treatment of Blepharitis Marginalis by Hydrogen Dioxid

**Published:** 1894-01

**Authors:** S. C. Ayres

**Affiliations:** Cincinnati, O.


### THE TREATMENT OF BLEPHARITIS MARGINALIS BY HYDROGEN DIOXID.

By S. C. AYRES, M. D., of Cincinnati, O.

The treatment of blepharitis marginalis is often unsatisfactory and disappointing. Relapses frequently occur, and remedies seem to lose their effect, and at times even to act unfavorably. It is true that much of our success depends on the faithfulness of the patient, or of his parents, in carrying out the treatment at home. Much also depends on the physical condition of the patient, and this should always receive careful attention. Anomalies of refraction seem to play an important rôle in perpetuating the disease, and these also should be carefully corrected. But aside from these considerations, the treatment is often prolonged, taxing the patience of the physician as well as of the client.

The remedies prescribed for the relief of this disease are numerous, and all have met with more or less success. During the past year I have used, with great satisfaction, hydrogen dioxid in the treatment of this disease. I was led to its use by some experiments in cases with suppurating rings round the cilia. After removing the crusts and applying the dioxid, there was a bubbling and boiling effect for a while, which soon subsided, leaving the ulcerated surface whitened, as if a solution of silver nitrate had been used upon it. The application was almost painless, and the lid was left clean and free from pus or scales.

These experiments were followed by a general adoption of this method in nearly all cases, but especially in those with ulcerations along the lid margin.

. . . . . . . . .

In the method advised, a clean remedy is used, which acts promptly and efficiently. By its chemic action it destroys the germs which cling so closely to the edges of the lids. It is not a cure-all or a specific, but I certainly have had the happiest results from its use.—*Medical News*, Dec. 23, 1893.

